# Efficacy confirmation study of aceneuramic acid administration for GNE myopathy in Japan

**DOI:** 10.1186/s13023-023-02850-y

**Published:** 2023-08-11

**Authors:** Madoka Mori-Yoshimura, Naoki Suzuki, Masahisa Katsuno, Masanori P. Takahashi, Satoshi Yamashita, Yasushi Oya, Atsushi Hashizume, Shinichiro Yamada, Masayuki Nakamori, Rumiko Izumi, Masaaki Kato, Hitoshi Warita, Maki Tateyama, Hiroshi Kuroda, Ryuta Asada, Takuhiro Yamaguchi, Ichizo Nishino, Masashi Aoki

**Affiliations:** 1 Department of Neurology, National Center Hospital, National Center of Neurology and Psychiatry (NCNP), Tokyo, Japan; 2 Department of Neurology, Tohoku University Graduate School of Medicine, 1-1 Seiryo-Machi, Aoba-Ku, Sendai, 980-8574 Japan; 3 Department of Neurology, Nagoya University Graduate School of Medicine, Nagoya, Japan; 4 Department of Clinical Research Education, Nagoya University Graduate School of Medicine, Nagoya, Japan; 5 Department of Neurology, Osaka University Hospital, Osaka, Japan; 6 Department of Neurology, Kumamoto University Hospital, Kumamoto, Japan; 7 Innovative and Clinical Research Promotion Center, Gifu University Hospital, Gifu, Japan; 8 Division of Biostatistics, Tohoku University Graduate School of Medicine, Sendai, Japan; 9 Department of Neuromuscular Research, National Institute of Neuroscience and Department of Genome Medicine Development, Medical Genome Center, National Center of Neurology and Psychiatry (NCNP), Tokyo, Japan

**Keywords:** GNE myopathy, Aceneuramic acid, Efficacy confirmation study, Ultra-orphan disease

## Abstract

**Background:**

A rare muscle disease, GNE myopathy is caused by mutations in the *GNE* gene involved in sialic acid biosynthesis. Our recent phase II/III study has indicated that oral administration of aceneuramic acid to patients slows disease progression.

**Methods:**

We conducted a phase III, randomized, placebo-controlled, double-blind, parallel-group, multicenter study. Participants were assigned to receive an extended-release formulation of aceneuramic acid (SA-ER) or placebo. Changes in muscle strength and function over 48 weeks were compared between treatment groups using change in the upper extremity composite (UEC) score from baseline to Week 48 as the primary endpoint and the investigator-assessed efficacy rate as the key secondary endpoint. For safety, adverse events, vital signs, body weight, electrocardiogram, and clinical laboratory results were monitored.

**Results:**

A total of 14 patients were enrolled and given SA-ER (n = 10) or placebo (n = 4) tablets orally. Decrease in least square mean (LSM) change in UEC score at Week 48 with SA-ER (− 0.115 kg) was numerically smaller as compared with placebo (− 2.625 kg), with LSM difference (95% confidence interval) of 2.510 (− 1.720 to 6.740) kg. In addition, efficacy was higher with SA-ER as compared with placebo. No clinically significant adverse events or other safety concerns were observed.

**Conclusions:**

The present study reproducibly showed a trend towards slowing of loss of muscle strength and function with orally administered SA-ER, indicating supplementation with sialic acid might be a promising replacement therapy for GNE myopathy.

*Trial registration number*: ClinicalTrials.gov (NCT04671472).

**Supplementary Information:**

The online version contains supplementary material available at 10.1186/s13023-023-02850-y.

## Introduction

GNE myopathy (also known as distal myopathy with rimmed vacuoles [DMRV], hereditary inclusion body myopathy [hIBM] or Nonaka Disease) is a hereditary myogenic disorder that results in muscle weakness due to a defect in muscle itself [1–6]. Although most myogenic disorders present with involvement of muscles in the trunk (around chest and hip) as well as upper arms and the proximal lower limbs, distal myopathies characteristically show gradually progressing weakness of muscles starting in the distal parts of the legs, particularly muscles such as tibialis anterior, with sparing of the quadriceps. GNE myopathy is also known as an ‘ultra-orphan’ disease: the estimated number of patients is just 150–400 in Japan and 40,000 worldwide [4, 7, 8]. Most patients with GNE myopathy present with manifestations at ages ranging from 15 to 35 [9].

Previously, the mechanism of onset of GNE myopathy was quite unknown. In 2001, Eisenberg et al. identified missense mutations in the *GNE* gene in patients with GNE myopathy [10]. The *GNE* gene encodes UDP-*N*-acetylglucosamine 2-epimerase/*N*-acetylmannosamine kinase (GNE/MNK) [11], the rate-limiting enzyme in the biosynthetic pathway of sialic acid. This finding suggested that the sialic acid required for muscle function is insufficiently supplied in the bodies of patients, due to a defect in its biosynthesis, and that replacement of sialic acid is a potential therapy to improve the pathology of GNE myopathy [5, 12]. Accordingly, the efficacy of replacement therapy was assessed in a GNE myopathy mouse model [13] established at the National Center of Neurology and Psychiatry (NCNP) in Japan. Continuous administration of aceneuramic acid to the model mice prior to disease onset allowed for comparable motor performance, contractile strength of skeletal muscles, biochemical parameters, and muscle pathological features over time, when compared to normal mice [14]. This non-clinical study suggested that oral supplementation of aceneuramic acid could be expected to improve the pathological condition and slow disease progression in patients with GNE myopathy.

To evaluate the safety and pharmacokinetics of orally administered aceneuramic acid in humans, we conducted phase I studies in patients with GNE myopathy [15] and observed elevated levels of serum free aceneuramic acid with no safety issues, using an extended-release formulation of aceneuramic acid (SA-ER). The results were consistent with those of a prior international phase II study [16]. In our recent phase II/III study in Japan (UMIN 000020683), the primary endpoint was change in the upper extremity composite (UEC) score (mean ± standard deviation [SD]), which was as small as − 0.1 ± 3.7 kg in the SA-ER group versus − 5.1 ± 3.4 kg in the placebo group [17]. The differences between the two groups were consistently statistically significant or nearly significant, when analyzed by several statistical methods. There were no adverse events that would pose safety concerns. In the United States, an international double-blind phase III study was conducted in GNE myopathy patients from seven countries, evaluating the efficacy and safety of SA-ER for 48 weeks. However, this study failed to demonstrate a significant difference between the SA-ER group and the placebo group for the primary endpoint, UEC score [18].

GNE myopathy is a rare disease that gradually progresses over a long time [5, 6, 19–21]. Furthermore, recent studies have suggested that the natural history of the disease may vary depending on patient background [20–22]. It is thus a challenge to clarify if replacement of sialic acid as a medical treatment would slow progression and improve symptoms of the disease. In the present phase III study, we focused on a subset limited by their GNE myopathy-Functional Activity Scale (GNEM-FAS) score and disease duration, to minimize variation in patient background and determine the reproducible efficacy of sialic acid supplementation in patients with GNE myopathy.

## Methods

### Study design

This was a phase III, randomized, placebo-controlled, double-blind, parallel-group, multicenter study conducted at five sites in Japan (Department of Neurology at Tohoku University Hospital, National Center Hospital, NCNP; Nagoya University Hospital; Osaka University Hospital; and Kumamoto University Hospital), in accordance with the Declaration of Helsinki, Good Clinical Practice guidelines, and study protocol. This study was reviewed in terms of ethical, scientific, and medical validity and approved by the institutional review board at each site before the start of the study. The study lasted from Feb 8, 2021 to March 29, 2022. The study is registered on ClinicalTrials.gov (NCT04671472).

### Participants

The key inclusion criteria for participating in this study included: confirmed mutations in the *GNE* gene and documented diagnosis of GNE myopathy; aged 20 years or more to 50 years or less at the time of consent acquisition; a GNEM-FAS score of 24 points or more in the upper extremity, and disease duration between 5 and 15 years, inclusive; those whose upper extremity muscle weakness had been confirmed based on manual muscle testing (MMT) or grip strength measurements over the past several years, or if they had participated in our previous phase II/III study, those who could demonstrate a decreased UEC score during the non-treatment period; and those who was able to provide reproducible force in their elbow extensors/flexors (i.e., two dynamometry force values with less than 15% variability in the dominant arm) at screening. The key exclusion criteria included: use of *N*-acetyl-d-mannosamine (ManNAc), sialic acid (ex., aceneuramic acid), or related metabolites, intravenous immunoglobulin (IVIG), or anything that can be metabolized to produce sialic acid in the body within 60 days before screening; hypersensitivity to aceneuramic acid or its excipients; and liver function test (i.e., aspartate aminotransferase, alanine aminotransferase, gamma-glutamyl transpeptidase) levels greater than 3 × the upper limit of normal (ULN) for age/gender, or serum creatinine of greater than 2 × ULN at screening.

### Randomization and intervention

Participants were randomly assigned in a 7:3 ratio to the SA-ER group and the placebo group, and all participants received either SA-ER or placebo. As in our recent phase II/III study, the investigational products used were an SA-ER tablet containing 500 mg of aceneuramic acid and a matching placebo tablet. Participants were administered four SA-ER or placebo tablets three times per day (6 g/day) orally, in the morning, early evening, and at bedtime after a meal (within 30 min after intake of food).

### Outcomes

Participants were subjected to the following efficacy assessments every 12 weeks, over 48 weeks. The primary endpoint was change in UEC score from baseline to Week 48. UEC score was calculated as the sum of bilateral average of strength values (in kilograms) for grip, shoulder abductors, elbow flexors, and elbow extensors. The strength of each muscle group was measured with a hand-held dynamometer.

The key secondary endpoint was efficacy rate based on a comprehensive assessment made by the investigator. First, the investigator assessed the following four items for one of the following: improvement, no change, deterioration, or undecidable (not applicable). This was for: (1) MMT for upper extremity, or grip strength, (2) UEC score, (3) change in UEC score compared with the placebo-administered GNE myopathy patients in previous clinical studies, and (4) other secondary endpoints. Subsequently, the comprehensive efficacy in each patient was assessed as effective (improvement judged by evaluator), ineffective (no improvement judged by evaluator), or unable to determine (The evaluator could not determine whether the drug was effective or ineffective) on integrating the assessments (1)–(4). If any one of the four assessment methods was “effective”, the subject was judged effective. The proportion of subjects judged as effective was defined as the effective rate.

Other secondary endpoints included the following changes from baseline to Week 48: GNEM-FAS upper extremity, mobility, and self-care scores; GNEM-FAS total score; bilateral average of individual muscle strength for grip, shoulder abductors, elbow flexors, and elbow extensors comprising UEC score; bilateral average of muscle strength for knee extensors.

The safety assessments included the incidence and severity of adverse events, vital signs and weight, electrocardiogram, and clinical laboratory tests.

### Serum free and total aceneuramic acid concentration

Methods for blood sampling and determination of serum free and total aceneuramic acid concentrations were performed according to those described in the previous study [15]. Serum free and total aceneuramic acid concentrations (µg/mL) were measured at baseline and every 12 weeks before administration (trough concentration).

### Sample size

According to data from the Registry of Muscular Dystrophy (REMUDY, http://www.remudy.jp/) [19], a registry system of patients with neuromuscular disease in Japan, the number of subjects included in this study was presumed to be not significantly different from that estimated at our previous phase II/III study in Japan. In the present study, eligible participants were required to meet additional inclusion criteria: (a) those who had a score of 24 points or more on the upper extremity for GNEM-FAS, and a disease period of 5 to 15 years; and (b) those whose upper extremity muscle weakness had been confirmed based on the results of MMT or grip strength measurements over the past several years, or if they had participated in our previous phase II/III study, those who were able to demonstrate a decrease in UEC score during the non-treatment period. Fourteen of 19 participants in the previous study met the criteria (a) [17]. Based on the current examination of the number of patients in the REMUDY and the five sites that had participated in the previous study, 28 patients were presumed to meet the inclusion criteria of the previous study. Accordingly, the number patients meeting the inclusion criteria in the present study was estimated to be 21 (= 28 × 14/19). Additionally, using the alternative criterion of “muscle weakness had been confirmed over the past few years prior to the present study” and the potential for small numbers of participants in placebo-controlled studies, the feasible target sample size was estimated to be 10–15 patients. This estimate included patients who had participated in the previous study; we thus sought to enroll an additional five or more patients in the present study.

### Statistical analysis

SAS software version 9.4 (SAS Institute Japan Ltd.) was used for statistical analyses. The following efficacy analyses were performed on the full analysis set (FAS) that included all the enrolled participants except for those with screening failure, no administration of the study drug, or no efficacy data after starting study treatment. *Primary endpoint*: for statistical analysis of change in UEC score from baseline to Week 48, we first constructed a linear mixed effect model including change from baseline as an objective variable, visit, treatment group and interaction of visit*treatment group as fixed effects, and subject as random effect. Using this model in consideration of intra-subject correlation, we estimated the least square mean (LSM) of the UEC change as well as between-group difference in LSM of UEC change and its 95% confidence interval [CI] by visit (Week 12, Week 24, Week 36, and Week 48). We also calculated mean ± SD for the change in UEC score by treatment group and visit, and further provided its transition diagram over time by treatment group. *Key secondary endpoint*: efficacy rate and its 95% CI for investigator-integrated efficacy assessment were estimated by treatment in the FAS. *Other secondary endpoints*: mean ± SD of change from baseline for each measurement was estimated by treatment group and visit, and its transition diagram over time was provided. For serum free and total aceneuramic acid concentrations, mean ± SD of measurement by treatment group and visit was calculated and its transition diagram over time was presented.

Safety assessments were performed in the safety population, which included all the participants who received the investigational drug and provided any safety data. Adverse events were coded according to the Medical Dictionary for Regulatory Activities, version 25.0. Severity was defined in accordance with the National Cancer Institute Common Terminology Criteria for Adverse Events (NCI CTCAE) ver. 5.0 (Japanese translation: Japan Clinical Oncology Group [JCOG] edition [CTCAE v5.0-JCOG]).

Further details on the Methods can be found in the full protocol provided in the Additional file 1.

## Results

### Baseline characteristics and disposition of patients

A total of 14 patients were enrolled and randomly assigned to the SA-ER group (10 subjects) or the placebo group (four subjects). All the participants in both groups received the assigned investigational agent for 48 weeks, completed the follow-up examination four weeks after the last administration, and were included in the FAS for efficacy assessments and safety population for safety assessments. No participant discontinued the study for any reason.

Table 1 shows baseline demographic and clinical characteristics of the patients. The median age of all the patients was 40.5 years and four patients (28.6%) were male. Overall, baseline characteristics including UEC score and GNEM-FAS upper extremity score were generally balanced between treatment groups.

**Table 1:** Demographic and other baseline characteristics (FAS)

	SA-ER	Placebo	Total
	(N = 10)	(N = 4)	(N = 14)
*Sex (n [%])*			
Male	3 (30.0)	1 (25.0)	4 (28.6)
Female	7 (70.0)	3 (75.0)	10 (71.4)
*Age (years)*^*a*^			
Mean (SD)	41.3 (6.1)	37.0 (6.9)	40.1 (6.4)
Median	40.5	39.5	40.5
Min, Max	32, 50	27, 42	27, 50
*Weight (kg)*^*b*^			
Mean (SD)	58.22 (10.18)	55.93 (14.84)	57.56 (11.12)
Median	56.90	49.05	52.75
Min, Max	48.3, 78.3	47.5, 78.1	47.5, 78.3
*Duration of illness (years)*^*c*^			
Mean (SD)	10.8 (2.6)	10.5 (2.6)	10.7 (2.5)
Median	10.5	11.0	10.5
Min, Max	6, 15	7, 13	6, 15
*Age of first GNE myopathy symptom (years)*^*d*^			
Mean (SD)	31.4 (6.0)	27.5 (5.1)	30.3 (5.8)
Median	30.5	28.5	30.5
Min, Max	20, 41	21, 32	20, 41
*UEC score at baseline (pattern: grip strength of 4 kg or less treated as 0 kg)*			
Mean (SD)	32.77 (12.37)	26.55 (8.74)	30.99 (11.50)
Median	27.53	27.03	27.53
Min, Max	19.60, 55.40	15.45, 36.70	15.45, 55.40
*GNEM-FAS upper extremity score*			
Mean (SD)	27.4 (2.8)	28.0 (2.2)	27.6 (2.5)
Median	26.5	27.5	27.0
Min, Max	24, 32	26, 31	24, 32
*Prior SA-ER administrated (n [%])*			
Yes	4 (40.0)	1 (25.0)	5 (35.7)

*GNEM-FAS* GNE myopathy-functional activity scale; *Max* maximum; *Min* minimum; *N* number of subjects in the treatment group; *SA-ER* sialic acid extended-release; *SD* standard deviation; *UEC* upper extremity composite

^a^Obtained informed consent

^b^Screening

^c^Duration of illness (years) = Year of informed consent—Year first symptom noted

^d^Age of first GNE myopathy symptom (years) = Year first symptom noted—Year of birth

### Outcomes

#### Serum concentration of aceneuramic acid

**Fig. 1:**
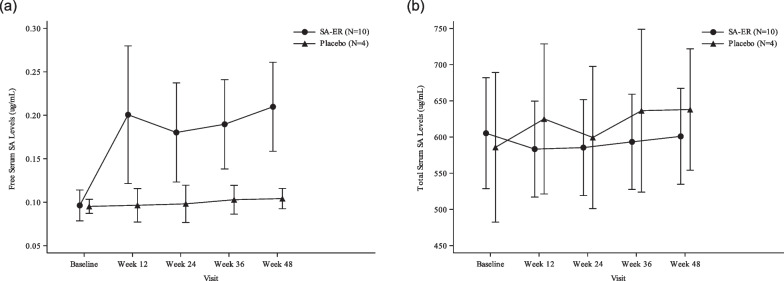
Mean and SD plots of serum free (**a**) and total **b** aceneuramic acid levels (FAS). Blood sampling and determination of serum free/total aceneuramic acid concentrations were performed as described previously [15]. FAS, full analysis set; SA-ER, sialic acid extended-release; SD, standard deviation

Serum free aceneuramic acid concentration was almost unchanged over time in the placebo group but clearly increased approximately twofold in the SA-ER group after Week 12 (Fig. 1). In contrast, serum total aceneuramic acid concentration did not change substantially in either group.

### Efficacy evaluations

#### Primary endpoint

**Fig. 2:**
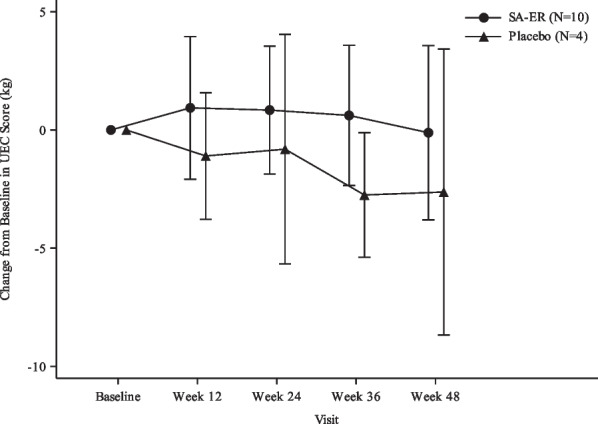
Mean and SD plots of change from baseline in UEC scores (FAS). Grip strength of 4 kg or less treated as 0 kg. FAS, full analysis set; SA-ER, sialic acid extended-release; SD, standard deviation; UEC, upper extremity composite

Figure 2 illustrates the change in UEC score from baseline to Week 48 in both groups. The decline in UEC score in the SA-ER group during the 48 weeks of treatment appeared to be suppressed, compared to the placebo group. We then constructed a linear mixed effect model to analyze the difference between the two groups. As shown in Table 2, the decrease in the LSM of the change in UEC score at Week 48 was numerically smaller in the SA-ER group (− 0.115 kg) compared to the placebo group (− 2.625 kg), with an LSM difference (95% CI) between the two groups of 2.510 (− 1.720 to 6.740) kg.

**Table 2:** Linear mixed effect model of change from baseline in UEC score (FAS)

Treatment group	Analysis visit	n	LSM	Standard error	Difference in LSM ^a^	95% CI for difference in LSM ^a^		
						Lower		Upper
SA-ER	Week 12	10	0.935	1.091	2.035	− 2.195	–	6.265
	Week 24	10	0.840	1.091	1.653	− 2.577	–	5.882
	Week 36	10	0.615	1.091	3.365	− 0.865	–	7.595
	Week 48	10	− 0.115	1.091	2.510	− 1.720	–	6.740
Placebo	Week 12	4	− 1.100	1.726	–	–	–	–
	Week 24	4	− 0.813	1.726	–	–	–	–
	Week 36	4	− 2.750	1.726	–	–	–	–
	Week 48	4	− 2.625	1.726	–	–	–	–

*CI* confidence interval; *FAS* full analysis set; *LSM* least square mean; *n* number of subjects analyzed; *SA-ER* sialic acid extended-release; *UEC* upper extremity composite

The linear mixed effect model includes treatment group, visit, treatment group*visit as fixed effects and subject as random effect. Degrees of freedom estimated using Kenward-Roger method. Not imputed, because there are not missing values at Week 48

^a^SA-ER—Placebo

#### Key secondary endpoint

For the investigator-assessed efficacy rate, outcomes in 7 of 10 subjects in the SA-ER group and two of four subjects in the placebo group were assessed as “effective.” A higher efficacy rate was noted with SA-ER (70%, 95% CI 34.75–93.33) as compared with placebo (50%, 95% CI 6.76–93.24) (Table 3).

**Table 3:** Efficacy rate for investigator's integrated efficacy assessment (FAS)

Treatment Group	N	n	Effective	Ineffective	Undeterminable	Efficacy Rate (%)	95% CI for Rate		
							Lower		Upper
SA-ER	10	10	7	3	0	70.0	34.75	–	93.33
Placebo	4	4	2	2	0	50.0	6.76	–	93.24

*CI* confidence interval; *FAS* full analysis set; *N* number of subjects in the treatment group; *n* number of subjects assessed; *SA-ER* sialic acid extended-release

#### Other secondary endpoints

**Fig. 3:**
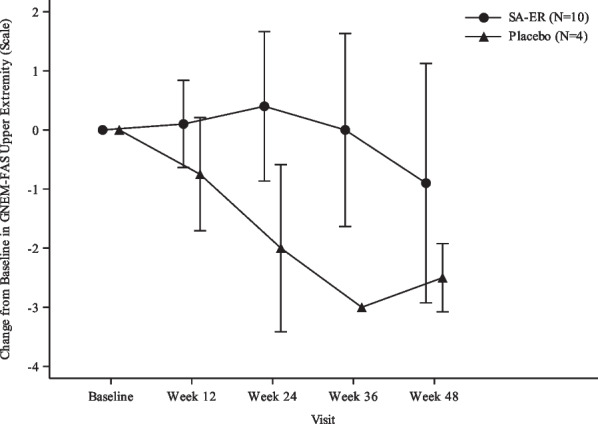
Mean and SD plots of change from baseline in GNEM-FAS upper extremity score (FAS). FAS, full analysis set; GNEM-FAS, GNE myopathy-Functional Activity Scale; SA-ER, sialic acid extended-release; SD, standard deviation

**Fig. 4:**
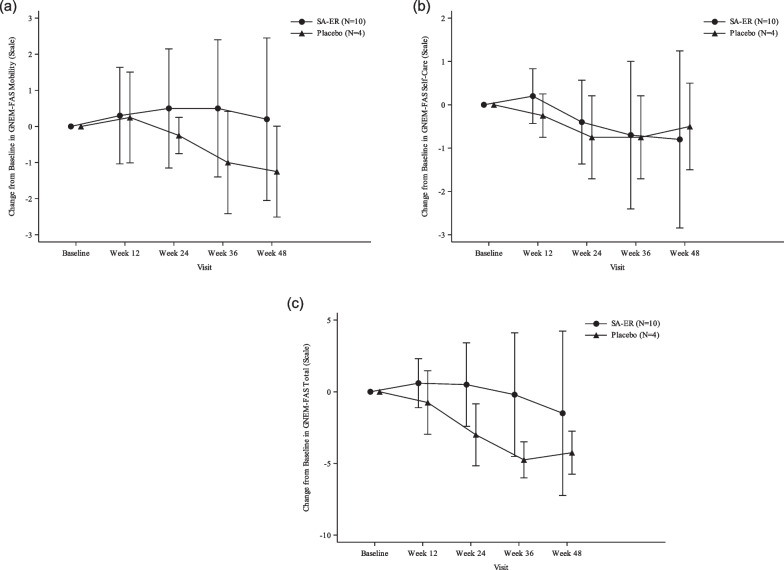
Mean and SD plots of change from baseline in other scores of GNEM-FAS (FAS). **a** mobility score, **b** self-care score, and **c** total score. FAS, full analysis set; GNEM-FAS, GNE myopathy-Functional Activity Scale; SA-ER, sialic acid extended-release; SD, standard deviation

GNEM-FAS upper extremity score did not change remarkably until Week 36 and was slightly decreased at Week 48 in the SA-ER group, in contrast to a clear decline at Week 12 and subsequently in the placebo group (Fig. 3). There was no obvious change in the GNEM-FAS mobility score over 48 weeks in the SA-ER group but there was an apparent decline at Week 24 and subsequently in the placebo group (Fig. 4a). The GNEM-FAS self-care score did not change substantially in either group (Fig. 4b). The GNEM-FAS total score exhibited a similar pattern to the GNEM-FAS upper extremity score (Fig. 4c). Regarding individual muscle strength related to grip, shoulder abductors, elbow flexors, and elbow extensors (comprising the UEC score), no decline was clearly seen in the placebo group, apart from grip strength; the ‘strength of any muscle’ in the SA-ER group remained unchanged (Additional file 1: Figure S1). Knee extensor strength as a measurement of lower extremity strength remained relatively spared in both groups over 48 weeks (Additional file 1: Figure S2).

#### Safety assessment

The number and incidence rate of reported adverse events were 55 events and 90% (9/10 subjects) in the SA-ER group, and 17 events and 100% (4/4 subjects) in the placebo group, respectively (Table 4). Serious adverse events reported comprised one event of COVID-19 in one subject in the SA-ER group and one event of papillary thyroid cancer in one subject in the placebo group (see Supplementary Table 1 for all adverse events). Both events were considered unrelated to the study drug. There were no deaths and no adverse events leading to study discontinuation in either group. Adverse events observed in two or more subjects of the SA-ER group were: dry eye, diarrhoea, gastrooesophageal reflux disease, pyrexia, immunisation reaction, and nasopharyngitis. Of these, diarrhoea, pyrexia, immunisation reaction, and nasopharyngitis were also reported in the placebo group. All immunisation reaction events were side reactions caused by vaccination against CoronavirusCOVID-19. Of the above events, just two were serious; all of the remaining events were mild or moderate in severity. Regarding gastrointestinal disorders, there were 19 events in six subjects in the SA-ER group and five events in two subjects in the placebo group. With respect to musculoskeletal and connective tissue disorders, there were eight events in two subjects in the SA-ER group but no events in the placebo group. Both types of disorders were reported more commonly with SA-ER as compared with placebo. No adverse events were considered related to the study drug.

**Table 4:** Summary of adverse events (safety population)

	SA-ER	Placebo
	(N = 10)	(N = 4)
	n (%) [Number of Events]	n (%) [Number of Events]
Any adverse events	9 (90.0) [55]	4 (100.0) [17]
Any adverse drug reactions	0 (0.0) [0]	0 (0.0) [0]
Serious adverse events	1 (10.0) [1]	1 (25.0) [1]
Serious adverse drug reactions	0 (0.0) [0]	0 (0.0) [0]
Adverse events leading to death	0 (0.0) [0]	0 (0.0) [0]
Adverse events leading to drug withdrawn	0 (0.0) [0]	0 (0.0) [0]
Adverse events occurring in 2 or more subjects		
Eye disorders	2 (20.0) [2]	0 (0.0) [0]
Dry eye	2 (20.0) [2]	0 (0.0) [0]
Gastrointestinal disorders	6 (60.0) [19]	2 (50.0) [5]
Diarrhoea	2 (20.0) [11]	1 (25.0) [1]
Gastrooesophageal reflux disease	2 (20.0) [2]	0 (0.0) [0]
General disorders and administration site conditions	2 (20.0) [2]	2 (50.0) [2]
Pyrexia	2 (20.0) [2]	2 (50.0) [2]
Immune system disorders	3 (30.0) [4]	1 (25.0) [1]
Immunisation reaction	3 (30.0) [4]	1 (25.0) [1]
Infections and infestations	3 (30.0) [5]	2 (50.0) [2]
Nasopharyngitis	2 (20.0) [3]	2 (50.0) [2]

*N* number of subjects in the treatment group; *MedDRA* medical dictionary for regulatory activities; *n* number of subjects with events; *SA-ER* sialic acid extended-release

Subjects with more than one event within a Preferred Term or System Organ Class are counted once for each category. Adverse events were encoded according to MedDRA Ver. 25.0

## Discussion

GNE myopathy is an orphan muscle disorder for which no treatment has been approved anywhere in the world [23]. Recently, we conducted a phase II/III study to evaluate the efficacy and safety of orally administered SA-ER in patients and showed a significantly higher UEC score at Week 48 in the SA-ER group compared to that in the placebo group, with no safety issues [17]. The aim of the present phase III study was to confirm the efficacy and safety of SA-ER in patients with GNE myopathy.

Consistent with observations from our previous phase I and phase II/III studies, and other clinical studies [15, 16, 18], we confirmed an obvious increase in serum free aceneuramic acid concentration after oral administration of SA-ER 6 g/day over 48 weeks, which indicated that orally administered aceneuramic acid was efficiently absorbed into the body and utilized in tissues.

In the present study, the primary endpoint was change in UEC score at Week 48, which was also employed as the primary endpoint in the international phase III study [18] and our recent phase II/III study [17], both following dosing with SA-ER. The decrease in the UEC score at Week 48 was numerically smaller in the SA-ER group than in the placebo group, thus indicating that oral administration of SA-ER 6 g/day but not placebo to patients with GNE myopathy might have maintained their muscle strengths as measured by UEC score during 48 weeks of treatment. This finding implied a trend toward a treatment benefit of SA-ER as compared with placebo. Subsequently, we performed a post hoc efficacy analysis using combined data of subjects in our recent phase II/III study [17] and newly enrolled subjects in the present phase III study, which showed statistically significant efficacy of SA-ER as compared with placebo (Additional file 1: Table S2). In addition, the results of the investigator-assessed efficacy rate also favored the effect of SA-ER on slowing disease progression. GNEM-FAS was developed to assess tatus of function, specifically in GNE myopathy patients, and has been used in previous aceneuramic acid supplementation studies [16, 18]. In our present study, plots of GNEM-FAS upper extremity and mobility scores separated from each other between the SA-ER group and the placebo group, indicating the effect of oral SA-ER on counteracting loss of muscle function. A similar plot separation was also observed in the GNEM-FAS total score, reflecting changes in upper extremity and mobility scores. Finally, knee extensor strength appeared stable over 48 weeks, probably due to the quadriceps-sparing nature of GNE myopathy [2, 9, 16].

Previously, the efficacy of SA-ER administration to patients with GNE myopathy was observed in the international phase II study [16] and our recent phase II/III study [17]. However, the international phase III study could not show a benefit of SA-ER as compared with placebo [18]. With regards to this, we assumed that the effect of SA-ER could be influenced in part by the amount of disease progression at baseline. The GNEM-FAS score assesses functional status in patients with GNE myopathy and appears to reflect progression of the disease more reliably than muscle strength. Using data from previous clinical studies including the international phase III study, we attempted to identify a subgroup showing the efficacy of SA-ER by limiting the baseline GNEM-FAS upper extremity score. Duration of disease was also considered, because patients with shorter disease duration are difficult to assess in terms of decline in muscle function, and patients with longer disease duration are unlikely to show large changes in disease progression. Finally, when focusing on a subgroup of patients who had a GNEM-FAS upper extremity score of 24 points or more and a disease duration of 5 years or more and 15 years or less, we noted significant efficacy (in change in UEC score) of SA-ER as compared with placebo in patients from the international phase II and phase III studies and our recent phase II/III study [17]. Based on this result, we conducted the present study with patients who met the above criteria and confirmed a clinical benefit of SA-ER, which suggests the importance of preserved muscle function and disease duration for efficacy evaluation of SA-ER treatment. In this context, we selected patients with deteriorating upper limb function, in order to detect the effect of SA-ER treatment during a limited period of evaluation.

Regarding safety, most of the adverse events were mild or moderate in severity. Two serious adverse events were reported but considered unrelated to the study drug. Thus, the safety profile of SA-ER is considered acceptable, which is consistent with previous phase I studies [15].

The present study has some limitations. First, owing to the rarity of GNE myopathy, a limited number of patients participated. Moreover, we added some inclusion criteria to minimize background variation in the study population. This decision may have affected statistical power to detect the difference between treatment groups. Because of the limited number of patients, a baseline deviation exists. Table 1 shows that the baseline body weight is lower in the placebo group, suggesting a higher degree of muscle atrophy at baseline. Additionally, the number of patients in the SA-ER group with prior SA-ER administration is different from the number in the placebo group. These baseline characteristics might have affected the results. Second, progression of GNE myopathy generally takes a long time and varies among individuals depending on the variable natural history of the disease. Despite the relatively homogeneous study population described above, muscle strength and function might not have declined in a similar manner in the absence of aceneuramic acid among participants during 48 weeks of treatment. Furthermore, long-term efficacy of SA-ER was not evaluated in the present study, although a long-term (72 weeks) extension study following our previous phase II/III study shows preservation of muscle strength (unpublished). Finally, only patients with mild symptoms (GNEM-FAS upper extremity score of 24 or more) were included in this study. Future studies should determine whether oral SA-ER is effective for patients with more severe symptoms.

In summary, the present phase III study in patients with GNE myopathy suggested a clinical benefit of orally administered SA-ER (6 g/day), with no safety concerns. Combined with our recent phase II/III study, oral aceneuramic acid is considered a promising therapeutic agent for mild GNE myopathy.

## Conclusions

The present study reproducibly showed a trend for orally administered SA-ER to slow loss of muscle strength and function, indicating supplementation with sialic acid may constitute promising replacement therapy for GNE myopathy.

### Supplementary Information

**Additional file 1**. **Figure S1**. Change from baseline in upper extremities. **Figure S2**. Change from baseline in knee extensors.

## Data Availability

All data provided are anonymized to respect the privacy of patients who have participated in the trial, in line with applicable laws and regulations.
