# Supplementary material for: Efficacy confirmation study of aceneuramic acid administration for GNE myopathy in Japan
Source: Orphanet J Rare Dis. 2023 Aug 11;18:241. doi: 10.1186/s13023-023-02850-y (PMC10416530; doi:10.1186/s13023-023-02850-y)
Supplement: Supplementary file 1 — Additional file 1. Figure S1. Change from baseline in upper extremities. Figure S2. Change from baseline in knee extensors. [file 13023_2023_2850_MOESM1_ESM.pdf]

Supplementary Figures

Supplementary Figure 1 Mean and SD plots in change from baseline in (a) grip, (b) shoulder abductors, (c) elbow flexors, and (d) elbow extensors (UEC parameter) (FAS). Grip strength of 4 kg or less treated as 0 kg. Elbow Ext, elbow extensors; Elbow Flex, elbow flexors; FAS, full analysis set; SA-ER, sialic acid extended-release; SD, standard deviation; Shldr Abd, shoulder abductors; UEC, upper extremity composite.

(a)

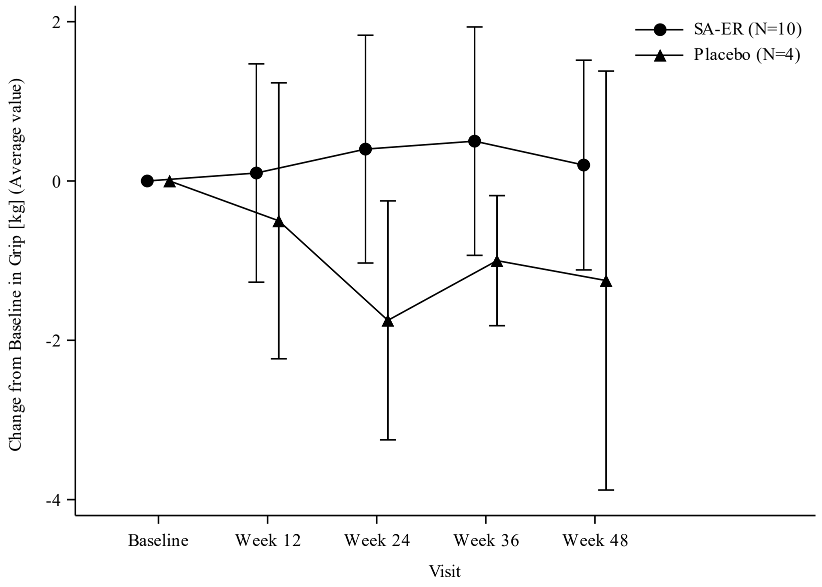

(b)

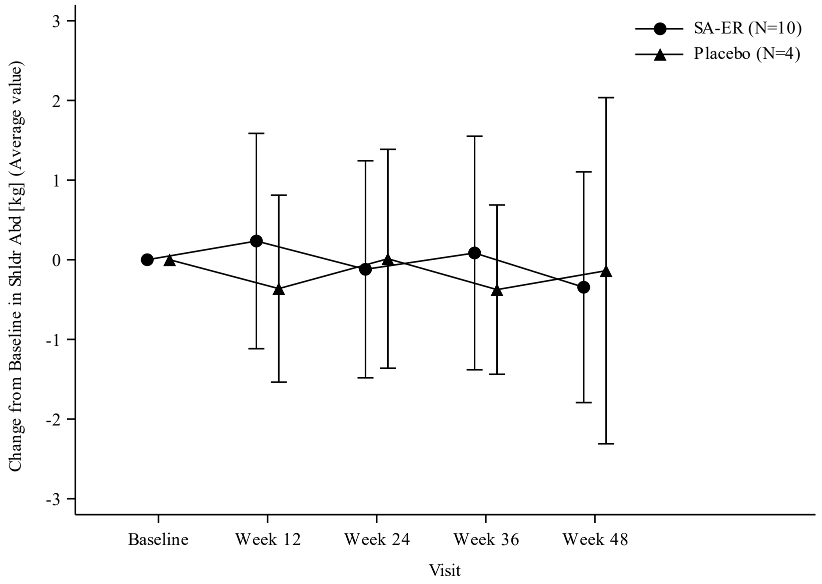

(c)

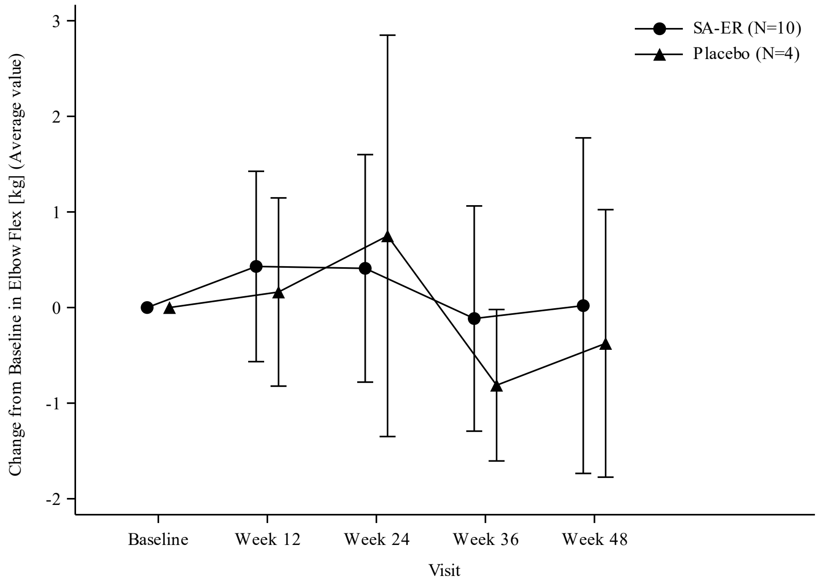
SA-ER (N=10)
Placebo (N=4)
Change from Baseline in Elbow Flex [kg] (Average value)
3
2
1
0
-1
-2
Baseline
Week 12
Week 24
Week 36
Week 48
Visit

(d)

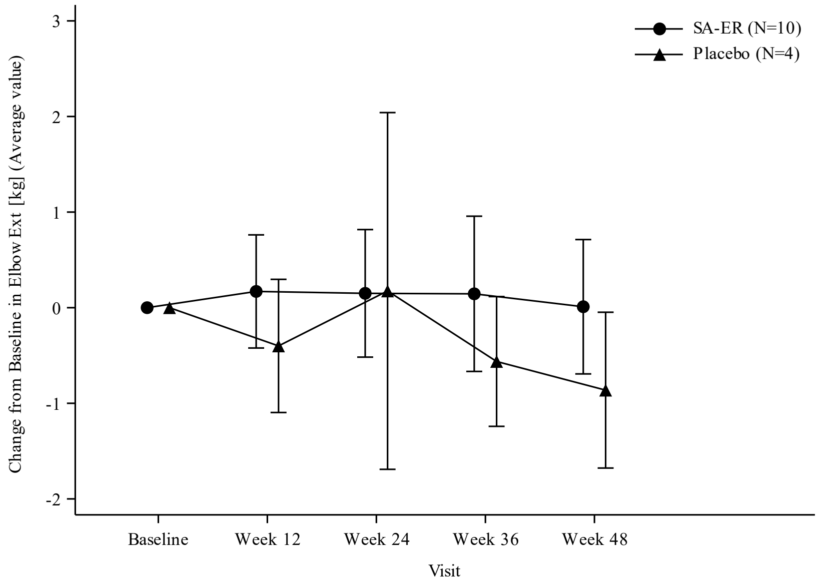
SA-ER (N=10)
Placebo (N=4)
Change from Baseline in Elbow Ext [kg] (Average value)
3
2
1
0
-1
-2
Baseline
Week 12
Week 24
Week 36
Week 48
Visit

Supplementary Figure 2 Mean and SD plots in change from baseline in knee extensors (FAS). FAS, full analysis set; Knee Ext, knee extensors; SA-ER, sialic acid extended-release; SD, standard deviation.

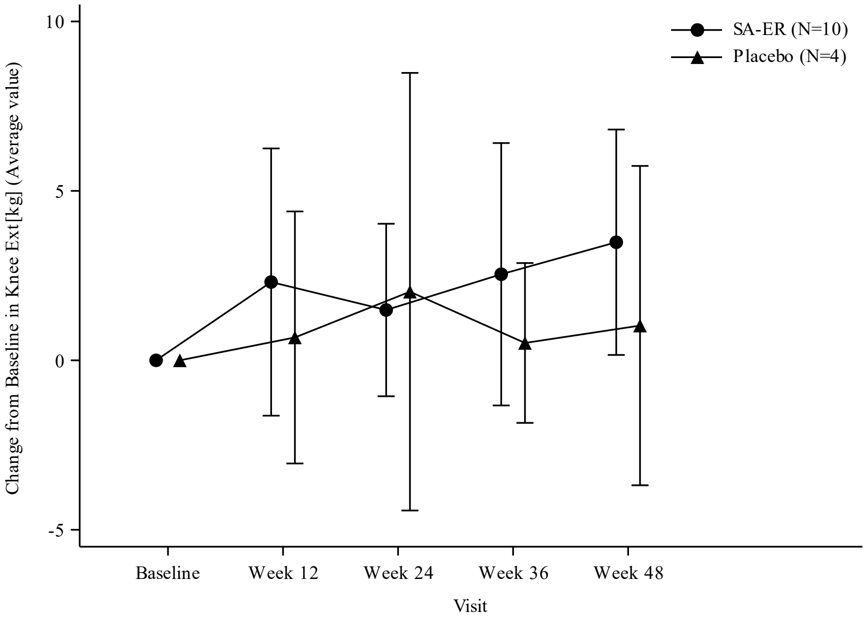

Supplementary Table 1 Full adverse events by system organ class and preferred term (Safety Population)

System Organ Class<br>Preferred Term	SA-ER<br>(N=10)<br>n (%) [Number of Events]	Placebo<br>(N=4)<br>n (%) [Number of Events]
Total	9 (90.0) [55]	4 (100.0) [17]
Ear and labyrinth disorders	1 (10.0) [1]	0 (0.0) [0]
Vertigo	1 (10.0) [1]	0 (0.0) [0]
Eye disorders	2 (20.0) [2]	0 (0.0) [0]
Dry eye	2 (20.0) [2]	0 (0.0) [0]
Gastrointestinal disorders	6 (60.0) [19]	2 (50.0) [5]
Abdominal pain	1 (10.0) [1]	0 (0.0) [0]
Abdominal pain upper	0 (0.0) [0]	1 (25.0) [1]
Constipation	1 (10.0) [2]	1 (25.0) [1]
Dental caries	0 (0.0) [0]	1 (25.0) [1]
Diarrhoea	2 (20.0) [11]	1 (25.0) [1]
Dyspepsia	0 (0.0) [0]	1 (25.0) [1]
Gastric polyps	1 (10.0) [1]	0 (0.0) [0]
Gastrooesophageal reflux disease	2 (20.0) [2]	0 (0.0) [0]
Nausea	1 (10.0) [1]	0 (0.0) [0]
Large intestine polyp	1 (10.0) [1]	0 (0.0) [0]
General disorders and administration site conditions	2 (20.0) [2]	2 (50.0) [2]
Pyrexia	2 (20.0) [2]	2 (50.0) [2]
Hepatobiliary disorders	1 (10.0) [1]	0 (0.0) [0]
Hepatic function abnormal	1 (10.0) [1]	0 (0.0) [0]
Immune system disorders	3 (30.0) [4]	1 (25.0) [1]

Immunisation reaction	3 (30.0) [4]	1 (25.0) [1]
Infections and infestations	3 (30.0) [5]	2 (50.0) [2]
Nasopharyngitis	2 (20.0) [3]	2 (50.0) [2]
Pharyngitis	1 (10.0) [1]	0 (0.0) [0]
COVID-19	1 (10.0) [1]	0 (0.0) [0]
Injury, poisoning and procedural complications	3 (30.0) [4]	2 (50.0) [3]
Facial bones fracture	1 (10.0) [1]	0 (0.0) [0]
Fall	0 (0.0) [0]	1 (25.0) [1]
Face injury	1 (10.0) [1]	0 (0.0) [0]
Contusion	0 (0.0) [0]	1 (25.0) [2]
Thermal burn	1 (10.0) [1]	0 (0.0) [0]
Skin abrasion	1 (10.0) [1]	0 (0.0) [0]
Investigations	1 (10.0) [1]	1 (25.0) [1]
Electrocardiogram ST segment depression	0 (0.0) [0]	1 (25.0) [1]
Gamma-glutamyltransferase increased	1 (10.0) [1]	0 (0.0) [0]
Musculoskeletal and connective tissue disorders	2 (20.0) [8]	0 (0.0) [0]
Arthralgia	1 (10.0) [1]	0 (0.0) [0]
Muscle spasms	1 (10.0) [5]	0 (0.0) [0]
Myalgia	1 (10.0) [1]	0 (0.0) [0]
Pain in extremity	1 (10.0) [1]	0 (0.0) [0]
Neoplasms benign, malignant and unspecified (incl cysts and polyps)	0 (0.0) [0]	1 (25.0) [1]
Papillary thyroid cancer	0 (0.0) [0]	1 (25.0) [1]
Nervous system disorders	1 (10.0) [1]	0 (0.0) [0]
Headache	1 (10.0) [1]	0 (0.0) [0]
Psychiatric disorders	0 (0.0) [0]	1 (25.0) [1]

Adjustment disorder	0 (0.0) [0]	1 (25.0) [1]
Skin and subcutaneous tissue disorders	2 (20.0) [6]	1 (25.0) [1]
Chloasma	1 (10.0) [1]	0 (0.0) [0]
Cold urticaria	1 (10.0) [2]	0 (0.0) [0]
Dermatitis	0 (0.0) [0]	1 (25.0) [1]
Eczema	1 (10.0) [3]	0 (0.0) [0]
Vascular disorders	1 (10.0) [1]	0 (0.0) [0]
Internal haemorrhage	1 (10.0) [1]	0 (0.0) [0]

MedDRA, Medical Dictionary for Regulatory Activities.

Subjects with more than one event within a Preferred Term or System Organ Class are counted once for each category.

Adverse events were encoded according to MedDRA Ver. 25.0.

Supplementary Table 2

Comparison of change in UEC score between groups (cFAS) (t-test)

	SA-ER group			Placebo group			Difference between groups		
Visit	N	mean	95% CI	N	mean	95% CI	mean	95% CI	p-value
Week 24	22	0.534	−0.951, 2.019	7	−2.921	−6.113, 0.270	3.456	0.453, 6.458	0.0257
Week 48	22	0.050	−1.648, 1.748	7	−4.507	−8.840, −0.174	4.557	0.964, 8.150	0.0148

CI, confidence interval; cFAS, combined full analysis set; N, number of subjects; SA-ER, sialic acid extended-release; UEC, upper extremity composite.

cFAS includes all the subjects in our previous phase II/III study and newly enrolled subjects in the present phase III study.
